# Peroxide of Hydrogen for Cleaning the Hands

**Published:** 1891-08

**Authors:** 


PEROXIDE OF HYDROGEN FOR CLEANING THE HANDS.—Noble (*Medical and Surgical Reporter*, Feb. 28, 1891) advises the following method fur rendering the hands aseptic:

The nails are trimmed reasonably short, and the subungual spaces cleared with the knife blade. The hands and forearms are then thoroughly washed in warm water, a good lather being made with soap, and a stiff nail-brush being vigorously applied. The water is renewed three times. The hands are next soaked in a saturated solution of oxalic acid. According to circumstances the finger tips are then soaked in peroxide of hydrogen. For the final bath, corrosive sublimate solution, one to one thousand, is employed. The hands remain in the sublimate solution three minutes.

